# Balanced forced‐diuresis as a renal protective approach in cardiac surgery: Secondary outcomes of electrolyte changes

**DOI:** 10.1111/jocs.15925

**Published:** 2021-08-19

**Authors:** Heyman Luckraz, Ramesh Giri, Benjamin Wrigley, Kumaresan Nagarajan, Eshan Senanayake, Emma Sharman, Lawrence Beare, Alan Nevill

**Affiliations:** ^1^ Department of Cardiothoracic Surgery, Consultant Cardiothoracic Surgeon American Hospital Dubai UAE; ^2^ Department of Cardiothoracic Anaesthesiology Consultant Cardiothoracic Anaesthesiologist, Heart & Lung Centre Wolverhampton UK; ^3^ Department of Cardiology Consultant Interventional Cardiologist, Heart & Lung Centre Wolverhampton UK; ^4^ Department of Cardiothoracic Surgery Heart & Lung Centre Wolverhampton UK; ^5^ Research Nurse, Research & Development Department Heart & Lung Centre Wolverhampton UK; ^6^ Department of Cardiothoracic Surgery, Chief Clinical Perfusionist University Hospitals of North Midlands Stoke‐on‐Trent UK; ^7^ Department of Statistics, Faculty of Education, Health, and Wellbeing University of Wolverhampton Walsall UK

**Keywords:** acute kidney injury, balanced forced diuresis, cardiac surgery

## Abstract

**Objectives:**

Forced‐diuresis during cardiopulmonary bypass (CPB) can be associated with significant electrolyte shifts. This study reports on the serum electrolyte changes during balanced forced‐diuresis with the RenalGuard® system (RG) during CPB.

**Methods:**

Patients at risk of acute kidney injury (AKI)—(history of diabetes &/or anaemia, e‐GFR 20–60 ml/min/1.73 m^2^, anticipated CPB time >120 min, Log EuroScore >5)—were randomized to either RG (study group) or managed as per current practice (control group).

**Results:**

The use of RG reduced AKI rate (10% for RG and 20.9% in control, *p* = .03). Mean urine output was significantly higher in the RG group during surgery (2366 ± 877 ml vs. 765 ± 549 ml, *p* < .001). The serum potassium levels were maintained between 3.96 and 4.97 mmol/L for the RG group and 4.02 and 5.23 mmol/L for the controls. Median potassium supplemental dose was 60 (0–220) mmol (RG group) as compared to 30 (0–190) mmol for control group over first 24 h (*p* < .001). On Day 1 post‐op, there were no significant differences in the serum sodium, potassium, calcium, magnesium, phosphate, and chloride levels between the two groups. Otherwise, postoperative clinical recovery was also similar.

**Conclusions:**

Balanced forced‐diuresis with the RG reduced AKI rates after on‐pump cardiac surgery compared to controls. Although the RG group required higher doses of IV potassium replacement in the postoperative period, normal serum levels of potassium were maintained by appropriate intravenous potassium supplementation and the clinical outcomes between groups were similar.

**Abbreviations**
AKI: acute kidney injury
CICU: cardiac intensive care unit
CIN: contrast induced nephropathy
CONSORT: Consolidated Standards of Reporting Trials
CPB: cardiopulmonary‐bypass machine
CVP: central venous pressure
e‐GFR: estimated‐glomerular filtration rate
i.v: intravenous
KDIGO: Kidney Disease: Improving Global Outcomes
KIDNEY: study Kidney protection using the RenalGuard® system in cardiac surgery
MAP: mean arterial pressure
NKCC2: sodium potassium chloride co‐transporter
OR: operating room
RG: RenalGuard® system
RIFLE: Risk, Injury, Failure, Loss of kidney function, End‐stage renal disease
R & D: Research & Development
SD: standard deviation
TAL: thick ascending limb

## 1 INTRODUCTION

The development of acute kidney injury (AKI) after cardiac surgery has been reported to be associated with significant postoperative morbidity and mortality both in the short‐term and the long‐term.^1^, ^2^ Many strategies have been described to reduce AKI^3^ and the use of the RenalGuard® system (RG) (RenalGuard Solutions Inc.) has recently been reported to reduce AKI rates after cardiac surgery.^4^

The principles and components of RG have been extensively described in previous reports.^4^, ^5^ In brief, it is a closed‐loop fluid management system which allows forced‐diuresis to be induced with low dose (0.25–0.5 mg/Kg) furosemide while inadvertent volume depletion is prevented by the administration of intravenous (i.v) fluids at a rate which can be matched in real‐time to the urine output. Its components include a high‐volume fluid pump, a high‐accuracy dual weight measuring system, a single‐use i.v set and a urine collection system that interfaces with standard Foley urinary catheter. There are, also, (a) real‐time displays of fluid replacement and urine volumes, (b) safety features such as automatic air and occlusion detection and (c) alerts to drain the urine bag or to replace hydration fluid bag. The RG console measures the volume of urine in the collecting set, then calculates urine flow rate and finally, infuses a pre‐set volume of hydration fluid to match the urine output, as decided by the treating physician. The console allows the user to set either an overall equal fluid balance (zero balance) or a net fluid gain above or loss below matched hydration as well as allowing the user to infuse fluid boluses.

It is well recognised that diuresis, especially by loop diuretics, is associated with electrolyte losses. The loop diuretics block the NKCC2 (sodium potassium chloride co‐transporter) channels present on the apical membrane of the thick ascending limb of the loop of Henle causing sodium and potassium excretion in the urine.^6^ Other effects of loop diuretics include metabolic alkalosis, hypocalcaemia, hypomagnesemia and hypochloraemia. Moreover, the use of the cardiopulmonary bypass machine (CPB) during cardiac surgery also causes significant volume and electrolytes shifts.^7^ These changes can lead to arrhythmias, cardiac dysfunction, brain oedema and in severe cases, neuronal demyelination.^8^ These effects are modulated due to alterations in cellular metabolism, cell membrane potentials and energy transformations. It is therefore imperative that the electrolytes levels are monitored during cardiac surgery and replenished appropriately and in a timely fashion.

This randomised control trial assessed the electrolyte changes (secondary outcomes) between patients treated with balanced forced‐diuresis (RG group) compared to controls, during cardiac surgery and within the first 24‐h post‐op, along with the clinical impact during the in‐hospital stay.

## 2 MATERIALS AND METHODS

### 2.1 Ethical approval

The KIDNEY study (**K** idney protect **i** on using the RenalGuar **d** ® system i **n** cardiac surg **e** r **y**) was reviewed and approved by the Institutional Research Committee (16CARD13) before seeking Ethical committee (16/NI/0246, 2nd December 2016) approval and was registered on ClinicalTrials.gov website (NCT02974946). The study was also supported by the National Institute of Healthcare Research, Clinical Research Network, United Kingdom (NIHR ID: 32769). All recruited patients gave written informed consent to partake in the study. Trial patients were treated according to the Declaration of Helsinki 2013.

### 2.2 Aims and objectives

The primary aim of the study assessed the impact of the RG system on the reduction of AKI (RIFLE—Risk, Injury, Failure, Loss of kidney function, End‐stage renal disease—criteria definition—50% increase in pre‐op “baseline” serum creatinine within first 3 days of surgery) in patients undergoing cardiac surgery. Baseline creatinine was defined as latest creatinine level available before surgery. Secondary aims included the electrolyte changes during surgery and within 24‐h post‐op as well as their impact on clinical outcomes during hospital stay. The primary outcome has already been published^4^ and the secondary objectives are being reported in this study.

### 2.3 Inclusion criteria

The inclusion criteria for recruited patients were one or more of the following: (i) diabetics (insulin or noninsulin dependent diabetes mellitus), (ii) eGFR of 20–60 ml/min/1.73 m^2^, (iii) Logistic Euroscore of 5 or above, (iv) haemoglobin level of 12.5 g/dl or below and (v) cardiac procedures when CPB time was likely to exceed 120 min.

### 2.4 Interventions

A research‐independent person based in the Research & Development department devised and managed a sealed opaque envelope system for randomisation of consented patients. In patients randomised to the RG system, balanced forced diuresis was started in anaesthetic room after patient intubation and continued throughout the cardiac procedure in the operating room (OR) and for up to 6 h after cardiac intensive care unit (CICU) admission. Forced‐diuresis was initiated with 20 mg bolus of IV furosemide. In some patients, the urine output rate was maintained by a furosemide infusion which was titrated to 10 mg/hour during surgery, with the aim of achieving a urine output of at least 200 ml/hour. This infusion was stopped in the OR at the end of procedure. In this study, patients were managed at a zero balance i.e. volume of urine output was matched in real‐time to the volume of Hartmann's fluid replacement infusion given through the RG system. On CICU, the RG system was continued for 6 h (matching Hartmann's i.v infusion rate to urine output rate). Additional furosemide bolus (20 mg i.v) was administered whenever the urine output fell below the 200 ml/hour threshold within the first 6‐h postadmission to CICU. Control group patients were managed as per current medical practice. They did not receive i.v furosemide in the OR. Otherwise, the management of the patients was similar including the anaesthetic technique and cardiopulmonary bypass run including the need for inotropic support to maintain a mean arterial pressure (MAP) >65 mmHg. CPB‐flow was calculated and maintained at a cardiac index of 2.4 l/min/m^2^. Bypass was performed at mild to moderate (32–34°C) hypothermia and patients were warmed to nasopharyngeal temperature of 36.5–37°C and bladder temperature of >35°C before discontinuation of CPB. The cardioplegia soution (Harefield Hospital formulation) had the following constituents: sodium—147 mmol/l, potassium—84 mmol/l, calcium—2 mmol/l, magnesium—80mmol/l, procaine—5 mmol/l and chloride 400 mmol/l. This was mixed with cold blood in a ratio 1:4 for myocardial protection during surgery.

### 2.5 Statistical analysis

To achieve the primary end‐point with a power of 0.8 and an alpha of 0.05, 110 patients per group were deemed adequate as per the power calculations. Categorical data are expressed as percentages and differences between the two groups assessed using the chi square (*χ* ^2^) test of independence. Continuous variables are expressed as median (minimum, maximum) or mean (*SD*) for skewed and Gaussian distributed data, respectively. Group comparison was carried out using two‐tailed *t* test or Mann–Whitney *U* test accordingly. Repeated measures were analysed via the generalised linear model for the changes in the variables (electrolytes, lactate, haemoglobin levels) across the various time points between the two groups and adjusted for any differences by the Bonferroni corrections. The tests were considered significant at *p* < .05. SPSS version 26.0 (IBM SPSS statistics 26) was used for statistical analyses.

Patients' recruitment was from 1st March 2017 to 4th September 2019. All patients' data were analysed on an intention to treat basis.

## 3 RESULTS

A total of 220 patients were randomised to the study (110 patients per group). Pre and intraoperative patients' data were not significantly different between the two groups (Table 1). The primary outcome of postoperative AKI was significantly lower in the RG group as compared to controls (10% vs. 20.9%, *p* = .025).

**Table 1:** Peri‐op characteristics of patients in the two groups

	RG group (*n* = 110)	Control group (*n *= 110)	*p* value
Age^a^, years	67.8 (9.3)	67.0 (9.2)	.33
Male (*n*, %)	87, 79%	84, 76%	.63
Nondiabetics (*n*, %)	30, 27%	30, 27%	.66
Pre‐op CVA (*n*, %)	11,10%	8, 7%	.63
Urgent surgery (*n*, %)	34, 31%	24, 22%	.13
STS mortality score^b^	0.96 (0.24, 6.63)	0.88 (0.20, 6.95)	.15
Pre‐op creatinine^a^, micromol/L	99.0 (28.2)	98.3 (34.7)	.29
Pre‐op haemoglobin^a^, g/L	135 (16)	134 (18)	.72
Operative procedures			.38
Isolated CABG (*n*, %)	53, 48%	59, 54%	
Isolated valve (*n*, %)	15, 15%	19, 18%	
Combined procedures (*n*, %)	40, 37%	31, 28%	
Day‐1 sodium level^a^, mmol/L	139 (3)	139 (2)	.35
Day‐1 potassium level^a^, mmol/L	4.7 (0.2)	4.7 (0.3)	.79
Day‐1 calcium level^a^, mmol/L	1.2 (0.3)	1.3 (0.4)	.06
Day‐1 magnesium level^a^, mmol/L	1.1 (0.2)	1.1 (0.2)	.41
Day‐1 phosphate level^a^, mmol/L	1.1 (0.2)	1.1 (0.2)	.21
Day‐1 bicarbonate level^a^, mmol/L	24 (2)	24 (2)	.92
Day‐1 chloride level^a^, mmol/L	105 (2)	105 (3)	.79
Day‐1 Haemoglobin^a^, g/L	99 (15)	102 (16)	.29

Abbreviations: CABG, coronary artery bypass surgery; CICU, cardiac intensive care unit; CVA, cerebro‐vascular accident; e‐GFR, estimated glomerular filtration rate; STS, Society of Thoracic Surgery.

^a^ Denotes mean (*SD*).

^b^ Denotes median (minimum, maximum).

The secondary aims of mean volumes of urine produced during surgery (2366 ± 877 ml vs. 765 ± 549 ml) and within first 24‐h post‐op on CICU (3310 ± 1303 ml vs. 2052 ± 804 ml) were significantly higher in RG group (*p* < .001).

**Figure 1:**
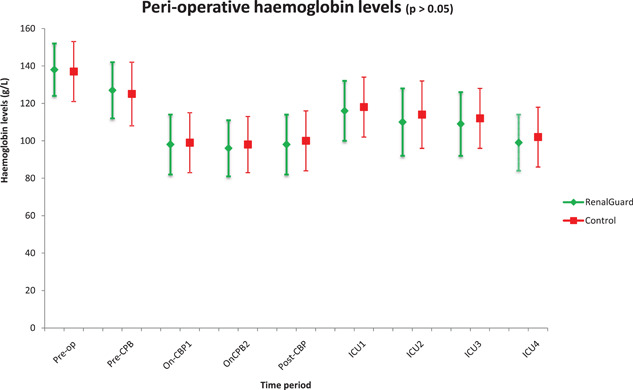
Haemoglobin levels in the perioperative period. Pre‐op: in anaesthetic room, Pre‐CBP: before initiation of cardiopulmonary bypass. On‐CPB1: on cardiopulmonary bypass for 15 min, On‐CPB2: before stopping cardiopulmonary bypass, Post‐CPB: poststopping cardiopulmonary bypass for 15 min. ICU1: on arrival to ICU, ICU2: on ICU for 6 h, ICU3: On ICU for 12 h, ICU4: On ICU for 24 h. ICU, intensive care unit

The median volumes of cold blood cardioplegia used were 2070 (995, 5381) ml and 1933 (1000, 5339) ml, respectively (*p* = .8). Patients in RG group received a median dose of 28 (0, 92) mg i.v furosemide in the OR. On the CICU 61% of study group (median dose 20 [0, 160] mg) and 53% of control group (median dose 20 [0, 180] mg) received i.v furosemide within the first 24 h of CICU admission (*p* = .99). There were no statistically significant differences between the groups in terms of the MAP and CVP during surgery and on the CICU. Similarly, there was no significant difference between (a) the haemoglobin levels during and after surgery (Figure 1) and (b) the postoperative blood transfusion rates (27% vs. 19%, *p* = .16), inclusive of intraoperative transfusions.

**Figure 2:**
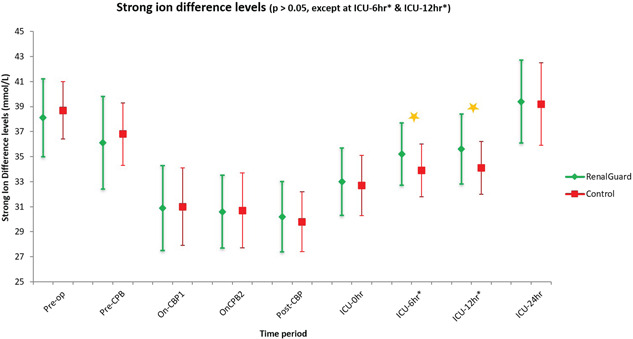
The strong ion difference levels (not significantly different except at ICU2* and ICU3* periods) at various time periods—Pre‐op: In anaesthetic room, Pre‐CBP: before initiation of cardiopulmonary bypass. On‐CPB1: on cardiopulmonary bypass for 15 min. On‐CPB2: before stopping cardiopulmonary bypass, post‐CPB: poststopping cardiopulmonary bypass for 15 min. ICU‐0 h: on arrival to ICU, ICU‐6 h: on ICU for 6 h, ICU‐12 h: on ICU for 12 h, ICU‐24 h: on ICU for 24 h. ICU, intensive care unit

**Figure 3:**
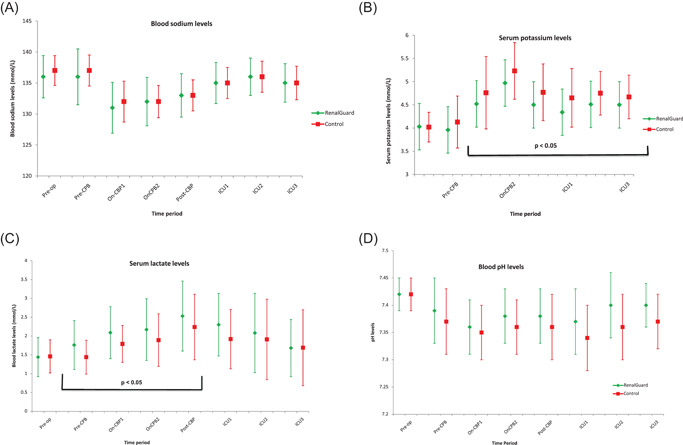
Electrolytes (A) sodium, (B) potassium, (C) lactate, and (D) pH levels in the perioperative period—pre‐op: in anaesthetic room, pre‐CBP: before initiation of cardiopulmonary bypass. On‐CPB1: on cardiopulmonary bypass for 15 min. On‐CPB2: before stopping cardiopulmonary bypass, post‐CPB: poststopping cardiopulmonary bypass for 15 min. ICU1: on arrival to ICU, ICU2: on ICU for 6 h, ICU3: on ICU for 12 h. ICU, intensive care unit

Patients in the study group required significantly higher doses of i.v potassium supplementation to maintain normal serum levels, with median potassium supplemental dose being 60 (0–220) mmol for RG as compared to 30 (0–190) mmol for control group over 24 h (*p* < .001). The serum potassium levels were maintained between 3.96 and 4.97 mmol/L for the RG group and 4.02 and 5.23 mmol/L for the controls. The changes in the strong ion difference ([Na^+^ + K^+^ + Ca2^+^ + Mg2^+^ levels] minus [Cl^−^ + lactate levels]) are shown in Figure 2. The changes in serum electrolytes (sodium, potassium), lactate and pH levels are depicted in Figure 3. On the first postoperative day, there were no significant differences in the sodium, potassium, calcium, magnesium, phosphate and chloride serum levels, between the two groups (Table 1).

There was no significant difference in incidence of atrial fibrillation rate, infections (chest, surgical site infection, sepsis), postoperative cerebro‐vascular events rate and median durations of CICU stays between the two groups. The median in‐hospital stay was 6 days for both groups.

One patient in RG group and two patients in control group died before hospital discharge. Causes of death were not related to the use of device and included cardiogenic shock, cardiac failure and sepsis (pneumonia) respectively.

## 4 DISCUSSION

The primary aim of this study was confirmed^4^ as AKI rate was reduced when the RG system was used perioperatively in cardiac surgery. The study also found that there were statistically significant electrolyte shifts during the use of the CPB and when it is combined with forced‐diuresis provided by the RG system. However, these changes did not impact on clinical outcomes.

Polderman reported electrolytes depletion in patients undergoing surgery with the CPB machine. The author suggested that these could be due to the intracellular shifts of electrolytes which are induced by hypothermia as well as urinary excretion of these electrolytes which was independent of the use of perioperative diuretics.^9^ The current study confirmed similar electrolytes shifts. However, clinically there was no significant difference in the patients' recovery as the levels of these electrolytes were closely monitored and supplementation of any electrolyte depletion was carried out in a timely manner.

Another important aspect which could influence electrolytes shifts is the acid–base status of the patient in particular, alkalosis.^7^ The latter is also assessed regularly during cardiac surgery and in the postoperative phase. There was no significant difference between the two groups in terms of bicarbonate and pH levels. Correction of acid–base disturbances can be through respiratory, renal or pharmacological routes.

The use of CPB circuit is recognized to lead to metabolic acidosis possible as a consequence of bicarbonate dilution.^10^ As per the Stewart calculations,^11^ metabolic acid–base status is a function of two independent variables interacting in intravascular and interstitial compartments: the strong ion difference and the concentration of nonvolatile weak acid. The former is the net charge of all fully dissociated ions (sum of Na^+^, K^+^ , Ca2^+^ , Mg2^+^ levels minus the sum of Cl^−^ and lactate levels). As for the weak‐acid, it consists of albumin and phosphate in the extracellular fluid, whereas within erythrocytes, it is primarily haemoglobin. An isolated increase in weak‐acid or a decrease in strong ion difference creates a metabolic acidosis. Changes in opposite directions, respectively, cause a metabolic alkalosis. In normal individuals not undergoing surgery a strong ion difference of around 40–45 mEq/L is considered normal^12^ whereas during a CPB run, a crystalloid strong ion difference of 24 mEq/L produces a balanced CPB with normal acid–base status.^13^ In this study the mean strong ion difference between the RG group and control group was similar at most time‐points except during the period of 6–12 h post ICU admission (Figure 2). Nevertheless, it was maintained between 30 and 45 mEq/L throughout the perioperative period.

The CPB circuit also causes significant haemodilution, hypomagnesaemia, and hyponatraemia. Haemodilution, as also discussed, causes acid–base changes. The degree of haemodilution can be minimized by using the retrograde autologous priming technique (RAP) where the priming volume is kept to around 1 L.^14^ This approach was used during this study and despite large volume diuresis and volume replacement in the RG group, the transfusion rate was similar between both groups as were the haemoglobin levels around the perioperative period. Precipitous hyponatraemia on CPB initiation can lead to osmotic demyelination syndrome causing cerebral injury and paralysis.^8^ Moreover, forced diuresis can exacerbate the degree of sodium ion loss. Thus it is important that the electrolytes are monitored appropriately. Last but not least, low magnesium levels have been reported during and after CPB.^15^ This can translate into clinical episodes of cardiac arrhythmias. In this study, both groups had similar and normal levels of magnesium.

### 4.1 Limitations

This was a single centre study. Moreover, as the study was not blinded, the management of the control group could have been influenced by some aspects of the Hawthorne effect.

## 5 CONCLUSION

In patients at‐risk for AKI, undergoing cardiac surgery with CPB, balanced forced‐diuresis as provided by the RG system significantly reduced the incidence of AKI. Compared to the control group, serum potassium levels were lower in the RG group. However, normal levels could be maintained by administration of IV potassium replacement in the postoperative period, thus maintaining similar clinical outcomes, with no adverse safety concerns.

## CONFLICT OF INTERESTS

The authors declare that there are no conflict of interests.

## AUTHOR CONTRIBUTIONS

*Concept and design*: Heyman Luckraz, Ramesh Giri, Benjamin Wrigley, Lawrence Beare, Alan Nevill. *Data analysis and interpretation*: Alan Nevill, Heyman Luckraz, Ramesh Giri, Benjamin Wrigley, Lawrence Beare. *Drafting article*: Heyman Luckraz, Ramesh Giri, Benjamin Wrigley, Kumaresan Nagarajan, Eshan Senanayake, Emma Sharman, Lawrence Beare, Alan Nevill. *Critical revision of article*: Heyman Luckraz, Ramesh Giri, Benjamin Wrigley, Kumaresan Nagarajan, Eshan Senanayake, Emma Sharman, Lawrence Beare, Alan Nevill. *Approval of article*: Heyman Luckraz, Ramesh Giri, Benjamin Wrigley, Kumaresan Nagarajan, Eshan Senanayake, Emma Sharman, Lawrence Beare, Alan Nevill. *Statistics*: Alan Nevill. *Funding secured by*: Heyman Luckraz, Ramesh Giri, Benjamin Wrigley. *Data collection*: Kumaresan Nagarajan, Eshan Senanayake, Emma Sharman, Lawrence Beare. *Project administration*: Heyman Luckraz, Ramesh Giri, Benjamin Wrigley, Emma Sharman.

## References

[1] Vives M , Hernandes A , Parramon F , et al. Acute kidney injury after cardiac surgery: prevalence, impact and management challenges. Int J Nephrol Renovasc Dis. 2019;12:153‐166. 31303781 10.2147/IJNRD.S167477 PMC6612286

[2] Mishra PK , Luckraz H , Nandi J , et al. Long‐term quality‐of‐life post acute kidney injury in cardiac surgery patients. Ann Card Anaesth. 2018;2:41‐45. 10.4103/aca.ACA_104_17 PMC5791486 29336390

[3] Joannidis M , Druml W , Forni LG , et al. Oudemans‐van Straaten HM, Schetz M. Prevention of acute kidney injury and protection of renal function in the ICU: update 2017: Expert opinion of the Working Group on Prevention, AKI section, European Society of Intensive Care Medicine. Intensive Care Med. 2017;43:730‐749. 28577069 10.1007/s00134-017-4832-y PMC5487598

[4] Luckraz H , Giri R , Wrigley B , et al. Reduction in acute kidney injury post cardiac surgery using balanced forced diuresis: a randomized, controlled trial. Eur J Cardiothorac Surg. 2020;59:562‐569. 10.1093/ejcts/ezaa395 PMC8043764 33236105

[5] Luckraz H , Giri R , Wrigley , Hennessy AM , Nicholas J , Nevill AM . The use of the RenalGuard system in cardiac surgery with cardiopulmonary‐bypass: a first in man prospective, observational, feasibility pilot study. Open Heart. BMJ. 2017;4:e000669. 10.1136/openhrt-2017-000669 PMC5640132 29071091

[6] Arumugham, VB , Shahin, MH . Therapeutic Uses Of Diuretic Agents. [Updated 2020 Jun 6]. StatPearls [Internet]. Treasure Island (FL): StatPearls Publishing, StatPearls; 2020. Jan ‐ Available from https://www.ncbi.nlm.nih.gov/books/NBK557838/ 32491770

[7] Liskaser F , Story DA , Hayhoe M , Poustie SJ , Bailey MJ , Bellomo R . Effect of pump prime on acidosis, strong‐ion‐difference and unmeasured ions during cardiopulmonary bypass. Anaesth Intensive Care. 2009;37:767‐772. 19775041 10.1177/0310057X0903700512

[8] Canaday S , Rompala J , Rowles J , Fisher J , Holt D . Chronic severe hyponatremia and cardiopulmonary bypass: avoiding osmotic demyelination syndrome. J Extra Corpor Technol. 2015;47:228‐230. 26834285 PMC4730166

[9] Polderman KH , Girbes AR . Severe electrolyte disorders following cardiac surgery: a prospective controlled observational study. Crit Care. 2004;8:R459‐R466. 15566592 10.1186/cc2973 PMC1065069

[10] Liskaser FJ , Bellomo R , Hayhoe M , et al. Role of circuit prime in the etiology and pathogenesis of cardiopulmonary bypass‐associated acidosis. Anesthesiology. 2000;93:1170‐1173. 11046201 10.1097/00000542-200011000-00006

[11] Stewart PA . Modern quantitative acid‐base chemistry. Can J Physiol Pharmacol. 1983;61:1444‐1461. 6423247 10.1139/y83-207

[12] Kellum JA . Determinants of blood pH in health and disease. Crit Care. 2000;4:6‐14. 11094491 10.1186/cc644 PMC137247

[13] Morgan TJ , Power G , Venkatesh B , Jones MA . Acid‐base effects of a bicarbonate‐balanced priming fluid during cardiopulmonary bypass: comparison with Plasma‐Lyte 148. A randomised single‐blinded study. Anaesth Intensive Care. 2008;36:822‐829. 19115651 10.1177/0310057X0803600611

[14] Kearsey C , Thekkudan J , Robbins S , Ng A , Lakshmanan S , Luckraz H . Assessing the effectiveness of retrograde autologous priming of the cardiopulmonary bypass machine in isolated coronary artery bypass grafts. Ann R Coll Surg Engl. 2013;95:207‐210. 23827293 10.1308/003588413X13511609956859 PMC4165246

[15] Parra L , Fita G , Gomar C , Rovira I , Marin J . Plasma magnesium in patients submitted to cardiac surgery and its influence on peri‐operative morbidity. J Cardiovasc Surg. 2001;42:37‐42. 11292903

